# Arrested human embryos are more likely to have abnormal chromosomes than developing embryos from women of advanced maternal age

**DOI:** 10.1186/1757-2215-7-65

**Published:** 2014-06-13

**Authors:** Shu-Tao Qi, Li-Feng Liang, Ye-Xing Xian, Jian-Qiao Liu, Weihua Wang

**Affiliations:** 1 Key Laboratory of Major Obstetrics Diseases of Guangdong Province, The Third Hospital Affiliated to Guangzhou Medical University, Guangdong, China; 2 Reproductive Medicine Center, the Third Hospital Affiliated to Guangzhou Medical University, Guangdong, China; 3 Houston Fertility Institute/Houston Fertility Laboratory, Houston, Texas, USA

**Keywords:** Aneuploidy, Arrested embryo, Blastocyst, Human

## Abstract

**Background:**

Aneuploidy is one of the major factors that result in low efficiency in human infertility treatment by in vitro fertilization (IVF). The development of DNA microarray technology allows for aneuploidy screening by analyzing all 23 pairs of chromosomes in human embryos. All chromosome screening for aneuploidy is more accurate than partial chromosome screening, as errors can occur in any chromosome. Currently, chromosome screening for aneuploidy is performed in developing embryos, mainly blastocysts. It has not been performed in arrested embryos and/or compared between developing embryos and arrested embryos from the same IVF cycle.

**Methods:**

The present study was designed to examine all chromosomes in blastocysts and arrested embryos from the same cycle in patients of advanced maternal ages. Embryos were produced by routine IVF procedures. A total of 90 embryos (45 blastocysts and 45 arrested embryos) from 17 patients were biopsied and analyzed by the Agilent DNA array platform.

**Results:**

It was found that 50% of the embryos developed to blastocyst stage; however, only 15.6% of the embryos (both blastocyst and arrested) were euploid, and most (84.4%) of the embryos had chromosomal abnormalities. Further analysis indicated that 28.9% of blastocysts were euploid and 71.1% were aneuploid. By contrast, only one (2.2%) arrested embryo was euploid while others (97.8%) were aneuploid. The prevalence of multiple chromosomal abnormalities in the aneuploid embryos was also higher in the arrested embryos than in the blastocysts.

**Conclusions:**

These results indicate that high proportions of human embryos from patients of advanced maternal age are aneuploid, and the arrested embryos are more likely to have abnormal chromosomes than developing embryos.

## Background

Human fertility, especially female fertility, declines dramatically in an age-dependent manner, mainly due to the decline of both quality and quantity of the oocyte and follicle pool with increasing maternal age [1,2]. The proportion of oocytes with abnormal chromosomes also increases with maternal aging, which is a major factor causing aneuploid formation in the resulting embryos. Embryonic aneuploidy is also a major cause of failed embryo implantation and miscarriage; hence aneuploidy is considered to be one of the most important factors causing the low efficiency of in vitro fertilization (IVF) treatments. Due to high rates of aneuploidy in the embryos from patients of advanced maternal age [3-7], several strategies have been carried out to select the euploid embryos for transfer so that the embryo implantation rate can be increased. One of these strategies is preimplantation genetic screening (PGS) by DNA microarray, which is able to examine all 23 pairs of chromosomes in the samples biopsied from embryos. It has been reported that significantly increased clinical pregnancy and embryo implantation rates were obtained after transfer of euploid blastocysts screened by DNA microarray [2,8,9].

Currently, PGS is performed in samples biopsied from polar bodies [10-13], cleavage embryos [14,15] or blastocysts [16,17]. It has been found that blastocysts have less mosaicism than cleavage embryos, thus most laboratories prefer blastocyst biopsy, in which multiple cells from the trophectoderm (TE) are biopsied and used for screening [8,9]. It is estimated that approximately 40-70% of human embryos produced by IVF are able to develop to blastocyst while others arrest at different earlier stages [18-22]. PGS is usually performed in the blastocysts, not in the arrested embryos because the information in the arrested embryos is of no clinical value. However, for better understanding of the mechanisms of embryo development and aneuploid formation, it is necessary to investigate the effects of chromosome integrity, in addition to embryo quality, on embryo development.

Previously, when fluorescence in-situ hybridization (FISH) technology was used for examination of 5-12 chromosomes in human embryos, it was found that a number of the arrested embryos were euploid [18], but it is still unknown whether these embryos are truly euploid or not. It was found that chromosome abnormalities occurred in any chromosome when embryos were examined by 24 chromosome microarray, and the proportion of abnormal 13, 18, 21, X and Y chromosomes (the most common chromosomes for FISH analysis) only accounted for 25% of all abnormities [2,23]. Previous studies also indicated that embryos screened by FISH technology had lower or similar implantation rates as compared with non-screened embryos [5,24,25]. These results indicate that the information obtained by FISH technology is not accurate to represent the chromosomal status of an embryo.

Due to the lack of information on the prevalence of chromosome abnormities in arrested human embryos, it is essential to examine all 23 pairs of chromosomes in the cohort of embryos produced from the same cycle in the patients so that the data can be compared directly between developing embryos and arrested embryos. The collected information would be useful to study the mechanism(s) by which some human embryos are unable to develop to blastocyst and arrest at different earlier stages and/or undergo fragmentation. Therefore, in the present study, experiments were designed to examine all chromosomes by DNA microarray in the blastocysts and arrested embryos in patients undergoing IVF and PGS.

## Methods

### Ethics

All patients undergoing IVF and PGS signed written consents for the laboratory manipulations and tests of the resulting embryos. This study was approved by the Institutional Review Board at The Third Hospital Affiliated to Guangzhou Medical University.

### Patient preparations for egg retrieval and PGS

Patients received PGS service because they were of advanced maternal age and needed aneuploidy screening of their embryos before transfer. For oocyte collection, patients were treated with a mixed protocol of human menopausal gonadotropin and a GnRH antagonist. The follicle stimulation hormone products (Follistim, Gonal-F, or Bravelle plus Menopure) were usually initiated within the first 2-3 days after the period began with a starting dose between 150 and 375 iu per day. The dose was adjusted as the stimulation progressed. Human chorionic gonadotropin (hCG) was injected at a dose of 5000-10000 units to induce final oocyte maturation when at least two dominant follicles reached a diameter of 18 mm. Eggs were retrieved via transvaginal ultrasound between 35–37 hours after hCG administration.

### Fertilization, embryo culture and embryo biopsy

Oocytes were cultured for 4-5 hours and the surrounding cumulus cells were removed in a HEPES-buffered medium containing 40 iu hyaluronidase, and the mature (metaphase II) oocytes were inseminated by intracytoplasmic sperm injection (ICSI). Fertilization was examined 16-18 hours after ICSI, and zygotes were cultured in a Global medium (http://IVFonline.com) supplemented with 10% serum protein substitute (SPS, IVFonline) at 37°C in a humidified atmosphere of 5.5% CO_2_, 5% O_2_ and balanced nitrogen until day 6 after insemination. On day 3, a hole of about 20 μm was opened on the zona pellucida with a laser generated by a ZILOS-tk laser system (Hamilton Thorne Bioscience Inc., NJ USA). On day 5, embryos were checked to see if a blastocyst had formed and if cells began to hatch from the opening in the zona pellucida. If some cells started to hatch, approximately three to five TE cells were biopsied using a 20-μm polished biopsy pipette with assisted cutting by laser. Blastocyst biopsy was performed on TE cells on day 5 or 6 depending on blastocyst development rate. The embryos that did not develop to blastocyst on day 6 were considered arrested, and up to five blastomeres were biopsied from these embryos. The biopsied cells were washed with a washing buffer, placed in tubes with cell lysis buffer and then frozen before being processed for microarray.

### Microarray with Agilent DNA array platform

The Agilent array platform was used to examine the chromosomes in the samples. The application of the Agilent DNA microarray platform in human embryos has been validated in the previous study, and the results were comparable to those obtained with other DNA microarray platforms, including the most common BluGnome platform [26,27]. Briefly, amplified samples were labeled with Cy3 using a SureTag DNA labeling kit. Labeled samples were then mixed with Cy5 control labeled samples. The labeled samples and controls were purified with a SureTag DNA labeling purification column, dried, dissolved in hybridization buffer containing Cot-1 DNA, 10 × blocking agent, and 2 × HI-RPM hybridization buffer, and loaded onto a SurePrint G3 60 K Oligo Microarray. After overnight hybridization at 65°C, microarrays were washed following Agilent washing protocol. Microarrays were scanned with a SureScan Microarray Scanner at 3 μM. Scanned images were analyzed by Cytogenomics software following Agilent comparative genomic hybridization (CGH) data analysis protocol.

### Statistical analysis

The data were analyzed by ANOVA and differences between groups were analyzed by chi-square test. P < 0.05 was considered statistically significant.

## Results

### High proportions of human embryos were aneuploid and the aneuploid rate increased with maternal age

**Figure 1:**
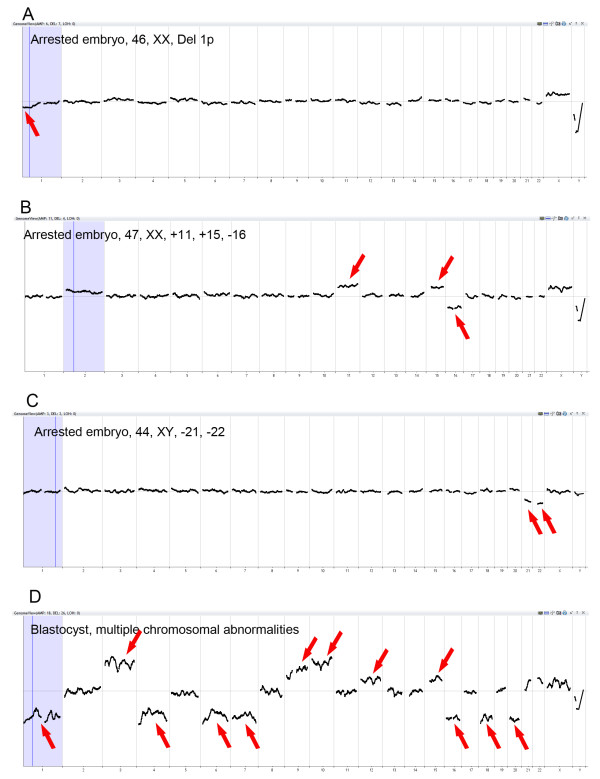
**PGS charts of human embryo samples with different types of chromosome abnormalities analyzed by the Agilent DNA array platform. (A)** An arrested embryo had euploid cells with partial chromosome deletion (46, XX, Del 1p). **(B)** An arrested embryo had multiple (47, XX, +11, +15, -16) chromosome errors. **(C)** An arrested embryo had multiple (44, XY, -21, -22) chromosome errors. **(D)** A blastocyst had multiple chromosomal abnormalities. Arrows indicate chromosome errors.

A total of 90 embryos from 17 patients were analyzed after biopsy and microarray. As shown in Table 1, 14 (15.6%) embryos were euploid with normal 23 pairs of chromosomes, and 76 embryos (84.4%) were either aneuploid with single or multiple chromosome abnormalities, or euploid with chromosomal deletion or duplication (Figure 1). We categorized these patients into two groups based on their maternal ages (36-39 and 40-45 years old). As shown in Table 1, the aneuploid rate (93.3%) in the patients aged 40 to 45 years was significantly (P <0.05) higher than that in the patients aged 36 to 39 years (75.6%).

**Table 1:** Microarray analysis of human embryos

	**Age (years)**		
**Observation**	**36-39**	**40-45**	**Total**
No. of cases	8	9	17
Total number of embryos biopsied and examined	45	45	90
No. (%)* of embryos with normal chromosomes	11 (24.4)^a^	3 (6.7)^b^	14 (15.6)
No. (%)* of embryos with abnormal chromosomes	34 (75.6)^a^	42 (93.3)^b^	76 (84.4)
No. (%)** of developing embryos (blastocysts)	25 (55.6)	20 (44.4)	45 (50)
No. (%)** of arrested embryos	20 (44.4)	25 (55.6)	45 (50)

*Percentages of embryos with normal (euploid) or abnormal (aneuploidy) chromosomes.

**Percentages of embryos developed to blastocyst stage or arrested at earlier stages.

^ab^P <0.05 within the same row.

### No significant difference was found in the developmental potential of embryos between younger patients and older patients

During IVF, approximately 50% of zygotes are able to develop to blastocysts while others (~50%) may arrest before blastocyst stage. When we analyzed the relationship between the developmental potential of embryos and maternal age, we found that the blastocyst rate (55.6%) was higher for the patients aged 36-39 years old than that of patients aged 40-45 years old (44.4%) (Table 1) but the difference was not statistically significant (P > 0.05, Table 1).

### More euploid embryos can develop to blastocysts than aneuploid embryos

**Figure 2:**
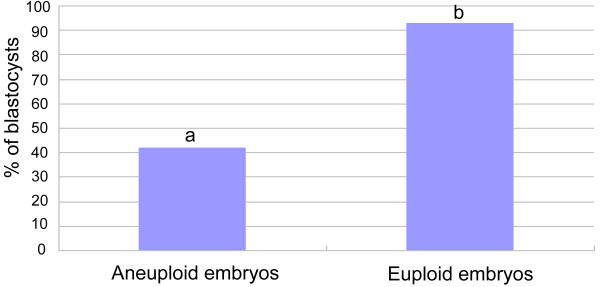
**The development of aneuploid and euploid embryos.** The rates of blastocysts and arrested embryos in aneuploid and euploid embryos were calculated respectively. Blastocysts were examined on day 5 and day 6 after fertilization, and chromosomes were examined by DNA microarray. ^ab^P < 0.01.

When the relationship between the developmental potential of embryos and their chromosome integrity was examined, it was found that 92.9% of the euploid embryos developed to blastocysts, while only 42.1% of the aneuploid embryos developed to blastocysts (Figure 2), which was significantly (P <0.01) lower than that from euploid embryos. This suggests that aneuploidy could significantly decrease the developmental potential of embryos.

### Arrested embryos are more likely to have abnormal chromosomes than developing embryos

**Figure 3:**
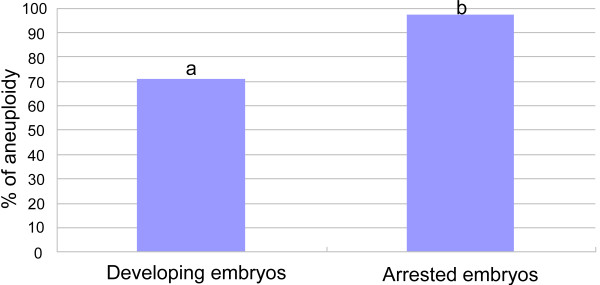
**The proportions of aneuploidy in the developing embryos and arrested embryos.** Embryo stages were examined on day 5 and day 6 after fertilization, and chromosomes were examined by DNA microarray. ^ab^P < 0.01.

**Figure 4:**
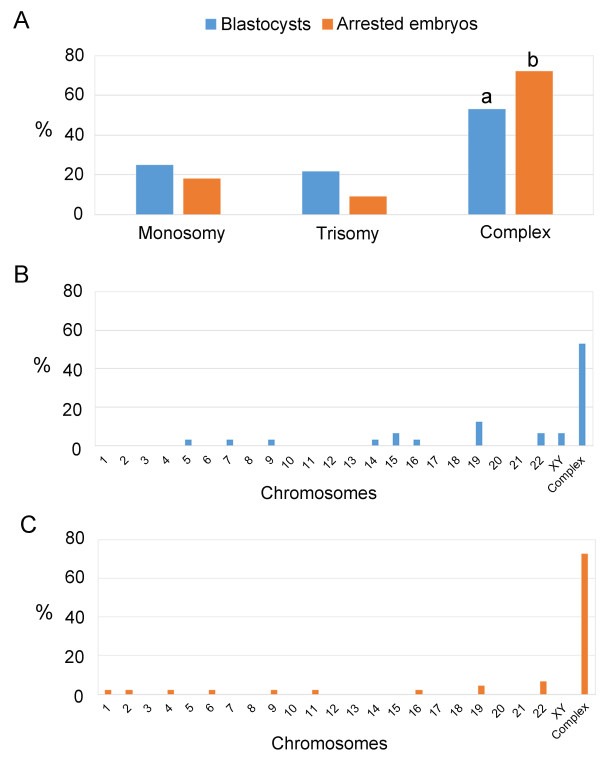
**Microarray results of abnormal chromosomes in blastocysts and arrested embryos. (A)** Abnormal chromosome distribution in the blastocysts and arrested embryos. Anomalies include monosomy, trisomy and complex (multiple) chromosome errors. **(B)** Distribution of chromosomal errors in the blastocysts. **(C)** Distribution of chromosome errors in the arrested embryos. ^ab^p < 0.05.

As aneuploidy significantly influences embryo development, we predicted that more arrested embryos would be aneuploid. Indeed, as shown in Figure 3, we found that 44 out of 45 arrested embryos (97.8%) were aneuploid, which was significantly (P <0.01) higher than the rate (71.1%) in the developing embryos (blastocysts) (Figure 3), indicating that arrested embryos are more likely to have abnormal chromosomes than developing embryos.When we further examined the chromosome distribution and the types of aneuploidies in the aneuploid embryos, as shown in Figure 4, we did not find significant differences in the chromosomal distribution between blastocysts and arrested embryos in terms of monosomy (25 vs 18.2%) and trisomy (21.9 vs 9.1%). However, more arrested embryos had multiple chromosomal abnormalities (complex: 72.3%) than blastocysts (53.1%). Errors occurred randomly in chromosomes, and there was no obvious difference between blastocysts and arrested embryos.

## Discussion

In the present study, we examined all 23 pairs of chromosomes in both blastocysts and arrested embryos from the same cycle in patients undergoing IVF and PGS by DNA microarray. The results indicate that almost all arrested embryos are aneuploid and the occurrence of aneuploidy in the blastocysts is closely related with maternal age.

It is well known that advanced maternal age is correlated with higher rates of embryonic aneuploidy [3-7]. In the present study, we found that a high proportion (84.4% in all embryos and 71.1% in blastocysts) of human embryos from patients of advanced maternal age was aneuploid, and the rates increased with maternal age. These results were consistent with those reported previously by others [2,3,5,7,8,22,23,28]. However, the aneuploid rate observed in the present study was higher than those reported in the previous studies [2,3,22,23]. One of the reasons for this high aneuploid rate in the arrested embryos may be due to the small sample size observed in the present study; thus the data may not represent larger populations of IVF patients. Another reason may be due to the embryo quality and patient population. The arrested embryos in the present study stopped development on day 3, but the biopsy and DNA array test were performed on day 6. Thus, the prolonged culture may have caused DNA degeneration or damages; this may also be an explanation of why more arrested embryos had multiple (complex) chromosomal abnormalities than blastocysts. Patient ages may be the major factor causing aneuploid formation in the present study. In a previous study with patients aged 37-46 years old, it was found that 55-80% of embryos were aneuploid when FISH probes were used [3]. We had a similar patient population in this study, but the aneuploid rate was higher than the results reported previously [3]. We examined all chromosomes but only 7 chromosomes were examined in the previous study [3]. Based on our previous study, chromosomal anomalies could occur in any chromosome, and the proportions of anomalies in the most common 5 chromosomes (13, 18, 21, X and Y) accounted for only 25% of total abnormal chromosomes. High aneuploid rates in human blastocysts were also reported in a recent study with 15,169 samples, in which the authors found that all samples could be aneuploid in 30-55% of the patients of advanced maternal age (42-45 years old) [28]. Taking into account these results, the high aneuploid rate observed in the present study should be related to the patients’ ages and embryo quality.

Aneuploidy could lead to reduced implantation and high miscarriage rates, but little is known about its mechanisms. Embryos usually arrest at various developmental stages for various reasons, such as culture conditions, patients’ ages and ovarian stimulation protocols. However, it is not known whether aneuploidy can directly influence embryo development. In the present study, we compared chromosome errors in the blastocysts and arrested embryos at various earlier stages and it was found that more euploid embryos developed to blastocyst than aneuploid embryos. These results indicate that aneuploidy can affect embryo development. We also found that not only more arrested embryos were aneuploid, but also more chromosomes had errors in the arrested embryos than in blastocysts. These results may explain the reasons that women of advanced age have lower embryo implantation rates, higher miscarriage rates and more birth defects, as compared to younger women.

Because maternal age increases the risk of aneuploidy, aneuploidy in turn affects implantation and causes miscarriage, PGS can be used to select embryos with normal chromosomes for transfer to improve implantation and pregnancy rates. However, previous FISH-based PGS did not increase the implantation and pregnancy rates due to its technical limitations (partial chromosome screening) [5,23-25]. DNA microarray technology has now replaced the FISH technology, as it can be used to examine all 23 pairs of chromosomes. It has been reported that transfer of euploid embryos screened by DNA microarray based PGS can significantly increase clinical pregnancy and embryo implantation rates [2,8,9]. The data obtained in the present study and previous studies clearly indicate that aneuploidy exists extensively in human embryos from patients of advanced maternal age, and PGS by DNA microarray is strongly recommended for these patients.

It was believed that the quality of embryos from younger patients may be better than those from older patients, so we assumed that the development rate of embryos from patients of advanced maternal age may be lower than that of younger patients. In the present study, we found that the blastocyst development rate was slightly higher in the younger patients than in the older patients. However, our data did not show a statistical difference between the two groups. This may be due to the patient population and small sample size in the present study. The data were mainly collected from older patients aged more than 37 years old in the present study. This population of patients is usually considered as the patients of advanced maternal age. Thus the younger patients in the present study may not represent the typical population of younger patients (less than 35 years old) undergoing IVF. Furthermore, the small sample size used in the present study may mask the differences, if such differences exist. Therefore, further studies with more samples from various ages of patients remain necessary for a clearer conclusion to be drawn. Currently, most clinics also offer blastocyst transfer to patients of advanced maternal age, but reduced embryo implantation still exists in these patients, indicating that aneuploidy could be the main reason affecting embryo implantation after transfer.

Many studies have been conducted in an effort to reveal the mechanisms of aneuploid origination in embryos from patients of advanced maternal age. It has been reported that maternal aging can dramatically influence the meiotic spindle assembly process in mammals [29-34], leading to spindle disorganization and chromosome segregation errors, which in turn cause aneuploid formation. In addition, the deterioration of sister chromatid cohesion [35-40] and failure of the spindle assembly checkpoint [41-44] in the oocytes are also crucial reasons for aneuploid formation.

## Conclusions

In conclusion, our present results demonstrate that high proportions of human embryos from patients of advanced maternal age are aneuploid, and the arrested embryos are more likely to have abnormal chromosomes than developing embryos. Most arrested embryos have multiple chromosome anomalies, indicating that the factors causing aneuploidy may also affect embryo development, but the detailed relationship between embryo development and aneuploidy needs further investigation.

## Abbreviations

IVF: In vitro fertilization; PGS: Preimplantation genetic screening; FISH: Fluorescence in-situ hybridization; hCG: Human chorionic gonadotropin; ICSI: Intracytoplasmic sperm injection; TE: Trophectoderm; CGH: Comparative genomic hybridization.

## Competing interest

We declare that we have no conflict of interest.

## Authors’ contributions

SQ and WW carried our study design and did the study. SQ, LL, YX, JL and WW did statistical analysis, and drafted manuscript. All authors read and approved the final manuscript.
